# Bronchogenic cyst associated with pericardial defect: Case report and review of the literature

**DOI:** 10.1186/1749-8090-6-85

**Published:** 2011-06-20

**Authors:** Andrea Imperatori, Nicola Rotolo, Elisa Nardecchia, Giovanni Mariscalco, Marco Spagnoletti, Lorenzo Dominioni

**Affiliations:** 1 Department of Surgical Sciences, Thoracic Surgery Unit, Varese University Hospital, University of Insubria, Varese, Italy; 2 Department of Surgical Sciences, Cardiac Surgery Unit, Varese University Hospital, University of Insubria, Varese, Italy

**Keywords:** Bronchogenic cyst, pericardial defect, video-thoracoscopy, harmonic scalpel

## Abstract

Partial defect of the pericardium combined with bronchogenic cyst is a very rare congenital anomaly. We describe the case of a 32-year-old man with a partial defect of the left pericardium and a bronchogenic cyst arising from the border of the pericardial defect. The cyst was successfully resected with the harmonic scalpel by three-port videothoracoscopic approach.

## Background

Mediastinal bronchogenic cysts (BC) are uncommon pathologic entities of congenital origin, representing 12% to 18% of all primary mediastinal masses [1-3]. Although BC are often asymptomatic, they can be complicated by infection, compression of the trachea or superior vena cava, intracystic hemorrhage, rupture, hemoptysis, and malignant changes [4-7].

BC have been reported to be also associated with other congenital malformations, including cardiac and pericardial anomalies [8-21]. Partial or total pericardial defect (PD) associated with BC is a very rare malformation, of which only 19 cases have been reported in the literature.

We present here a case of mediastinal BC associated with partial PD, successfully treated by a video-assisted thoracoscopic surgery (VATS). We also review the literature concerning the clinical presentation and management of BC associated with PD.

## Case presentation

**Figure 1:**
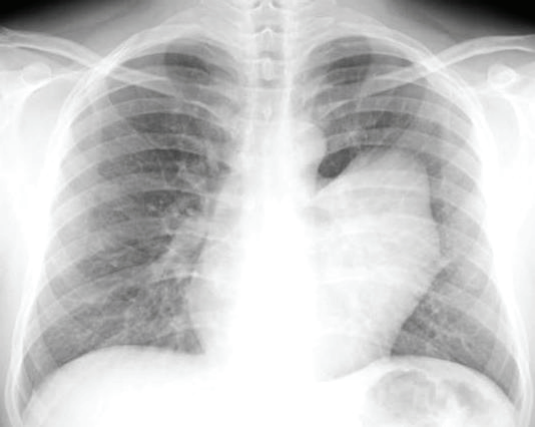
**Chest X-rays showing mediastinal mass**. Chest X-ray showing a large round opacity of the left hilum.

**Figure 2:**
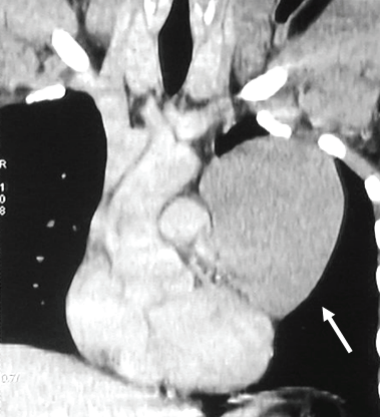
**CT scan showing cystic mass**. CT scans showing a well circumscribed cystic mass (10 × 10 cm) adjacent to the left pulmonary artery (arrow).

A 32-year-old man was admitted to our department complaining of left chest pain and cough. Chest radiography showed a large round opacity (10 × 10 cm) of the left hilum (Figure 1). The electrocardiogram was normal. Computed tomography (CT) scan demonstrated a large cystic mass arising from the pericardium, adjacent to the left pulmonary pericardial sinus (Figure 2); no other abnormalities were observed.

**Figure 3:**
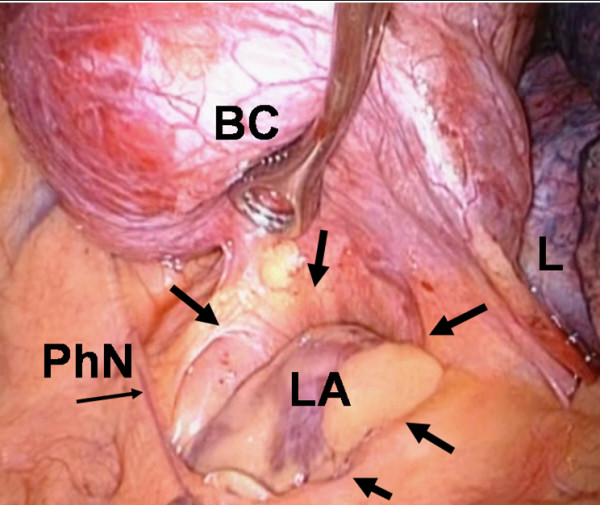
**Intraoperative video-thoracoscopic detail**. Intraoperative view showing bronchogenic cyst (BC), left atrial appendage (LA) visible through the pericardial defect (arrows), phrenic nerve (PhN), left upper lobe of the lung (L).

**Figure 4:**
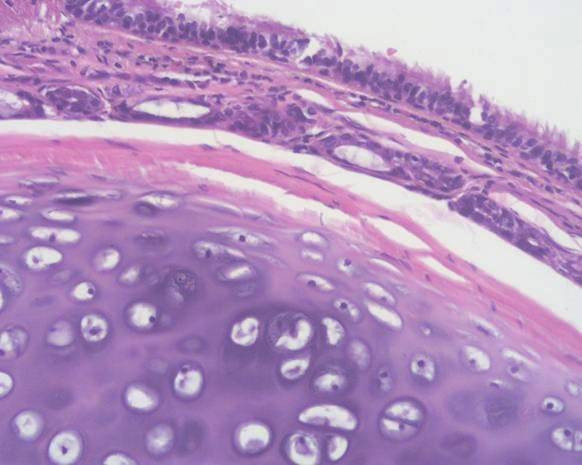
**Histology of cystic mass**. Histological section of the bronchogenic cyst, showing ciliated epithelium and cartilages (HE stain, 20X)

Resection of the cystic mass was effected by VATS, with three-port approach on the left side. At thoracoscopy a left upper pericardial defect (3 × 4 cm), oval-shaped, was found. A large cyst was identified, arising from the upper border of the PD. The cyst was adherent to the left main pulmonary artery and to the visceral pleura of the left lung upper lobe (Figure 3). After needle aspiration of part of the dense fluid content of the cyst, the latter was dissected from adhesions to the lung and to the upper border of the pericardial defect, using the harmonic scalpel. The cyst was radically resected with minimal blood loss and without complications. The left atrial appendage was partly bulging from the pericardial defect, but without herniation. Therefore the defect was left untreated. Pathology of the resected specimen revealed a bronchogenic cyst (Figure 4). The postoperative course was uneventful. Cardiac function was monitored postoperatively by transthoracic echocardiography, which demonstrated no cardiac herniation, and the patient was discharged on the 5th postoperative day. At 18-month follow-up the patient was asymptomatic; cardiac magnetic resonance imaging and transthoracic echocardiography demonstrated no cardiac herniation nor functional deficiency, also when the patient was examined in the left lateral decubitus position [22].

## Discussion

BC are the most common cystic lesions of the mediastinum and account for 18% of all primitive mediastinal masses. The prevalence of BC is difficult to ascertain, because they frequently are asymptomatic [5,23-26]. While most BC are located in the mediastinum, 15% to 30% of them are found within the lung parenchyma; in the latter case the lower lobes are most commonly involved [1-3]. Atypical locations of BC are also reported, including the neck, the spinal dura mater and the diaphragmatic region [1-3]. BC are congenital malformations arising from the primitive foregut with an abnormal division of the tracheobronchial tree; the stage of embryonic development determines the mediastinal location [26]. In case of early separation from the main tracheobronchial tree, BC are located in the mediastinum close to the trachea, carina, main bronchi or esophagus; histologically these entities present ciliated epithelium derived from either the respiratory or the alimentary tract. When the separation occurs late, BC involve the lung parenchyma and the cysts present a lined respiratory epithelium [5,27]. BC are most frequently unilocular; their fluid content may be clear, or dense and yellow, or hemorrhagic, or mixed with air in case of intrapulmonary location of the cyst.

No clinical presentation is specifically suggestive of BC because these lesions are frequently asymptomatic, their diagnosis being incidental [6,23]. Chest pain, cough, dyspnea, and dysphagia are reported as possible clinical manifestations of BC, arising from compression of the esophagus and/or major airways [4-7,23]. In the case we are reporting, the patient was symptomatic for cough and chest pain; the preoperative diagnosis of BC was made by chest radiography and CT scan.

Complete surgical excision is the treatment of BC generally accepted, because these lesions do not spontaneously regress and can enlarge or become infected. Several surgical techniques have been described [5,23-26,28]. Drainage of a compressive cyst is a temporary palliative procedure, generally reserved to inoperable patients, to the management of recurrences and of severe compression [5,29]. Surgical approaches include thoracotomy and VATS [5,23-26]. In the last decade VATS has emerged for the treatment of BC in absence of severe adhesions to surrounding mediastinal organs [29,30]. In the present case the mediastinal BC was resected by thoracoscopic approach, using the harmonic scalpel, a technique that has become available in recent years and proved to be safe and effective [31]. Harmonic scalpel confers some advantages over conventional methods of dissection, such as electric cautery, in VATS procedures. It reduces blood loss, duration of drainage and length of the VATS procedure with a comparable cost as compared to electric cautery. Similar advantages of harmonic scalpel have been observed in other surgical fields, such as thyroid surgery [32], video-assisted thoracoscopic thymic resection [33] and vascular surgery [34].

Various congenital anomalies of the heart, lung, chest wall and diaphragm have been reported to be associated with BC. PD, patent ductus arteriosus, atrial septal defect, tetralogy of Fallot, mitral stenosis, pulmonary sequestration and diaphragmatic hernia have been encountered in association with BC [4,5]. During development of the pleuropericardial fold, pericardial defects and lung anomalies such as bronchogenic cyst may occur together [15]; this event is unlikely to be co-incidental. In the present case, a partial PD was incidentally discovered during surgery for resection of BC. Congenital PD is a rare anomaly presenting as a complete or partial absence of the pericardium. Partial absence more commonly occurs on the left side (70%) than on the right (17%) or in the inferior portion of the pericardium [22]. The prevalence of PD is likely underestimated, because the symptoms are absent or scarce and the diagnostic criteria are poorly known [35]. It should be emphasized that the intact pericardium over the left atrial appendage is very thin and may not be identified even in normal people; thus, a partial left PD is nearly impossible to be recognized by routine CT scan, unless the atrial appendage is frankly bulging from the defect [22].

Usually patients with PD are asymptomatic and the diagnosis is incidental during thoracic surgery for unrelated conditions, as in our case.

Atypical angina symptoms or dyspnea are possible unspecific manifestations [36]. Although small defects occasionally induce serious or even lethal complications due to the incarceration of cardiac tissue, large or total left-sided PDs are usually considered benign and deserve no treatment. Surgical repair is required in case of large cardiac herniation and imminent strangulation [36].

We reviewed the literature pertinent to BC associated to PD, and found only 17 published cases of that combined congenital malformation. Table 1 summarizes the features of the 14 cases for which complete information were available (3 cases were not reported in English language) and shows that all patients had symptoms due to BC, while the PD was incidentally discovered during surgery performed to excise the cyst; left partial PD predominated, and in only 3 cases a direct suture of the defect was required.

**Table 1:** Cases of bronchogenic cyst associated with pericardial defect published in the English language literature

Author [ref]	Year	Case n.*	Age/Sex	Size (cm)	Location	Symptoms	Surgical Treatment†	Pericardial defect Location/Extension (cm)	Other congenital anomalies
Rusby and Sellors [12]	1945	1	19, F	6	L upper lobe	Chest pain	Excision	L, Partial	none
Jones P. [13]	1955	2	9, M	-	L hilum	Bronchitis	Excision	L, Partial 3 × 2	none
		3	22, M	-	L upper lobe	Asymptomatic	Lobectomy	L, Partial (extensive)	none
		4	21, M	-	R lung apex	Asymptomatic	Lobectomy	R, Partial 4 × 2.5	none
		5	22, F	-	L upper lobe	Asymptomatic	Excision	L, Partial, 2.5 × 2.5	none
		6	9, M	10 × 6	R lung apex	Infections	-	R, Partial	Double BC, vascular
Warner et al. [14]	1958	7	7 1/2, M	2 × 2	L upper lobe	Asymptomatic	Excision	Complete	Pleural defect
Hamilton LC. [15]	1961	8	10, M	-	L lower lobe	Pneumonia	Excision	L, Partial	Pleural defect
Mukerjee S. [16]	1964	9	24, F	-	L upper lobe	Chest pain	Excision	L, Partial	Pleural defect
Kwak et al. [17]	1971	10	15, F	7.5 × 5 × 3.6	L hilum	Asymptomatic	Excision + PDS	L, Partial 2 × 2	none
Kassner et al. [18]	1975	11	2, F	6 × 6	L upper lobe	Asymptomatic	Excision + PDS	L, Partial 2 × 2	Hip dislocation
Victor and Daniel [19]	1981	12	14, M	5 × 4	R upper lobe	Dyspnea, dysphagia	Excision + PDS	R, Partial	none
Eom et al. [20]	2007	13	18, M	8 × 7 × 4.5	L mediastinum	Cough, dyspnea	Excision	L, Partial	none
Özpolat et al. [21]	2009	14	15, M	-	L upper lobe	Chest pain, dysphagia, dyspnea	Excision	L, Partial	ASD, MVP, hypospadias
*Present case*	*2010*	*15*	*32, M*	*10 × 10*	*L hilum*	*Chest pain, cough*	*Excision (VATS)*	*L, Partial*	*none*

ASD, atrial septal defect; BC, bronchogenic cyst; F, female; L, left; R, right; M, male; MVP, mitral valve prolapse; PDS, pericardial defect sutured; VATS, video-assisted thoracoscopic surgery.

* Details of three other cases were not listed because publications were not in English language [ref. # [23-25]].

† Surgical approach to the cysts was accomplished in all case by left or right thoracotomy.

To our knowledge, the case of BC associated to PD presented here is the first described that was treated by VATS approach, using the harmonic scalpel for resecting the cyst. The decision to close the PD can only be made on an individual basis, after evaluation of the specific anatomical alterations. In our case we did not close the partial PD because the left atrial appendage was adherent with the inner aspect of the pericardium and did not herniate; that decision proved to be appropriate, because at 18-month follow-up the transthoracic echocardiography confirmed no cardiac herniation nor functional deficiency.

In conclusion, in the case presented the VATS approach to resect the BC with the harmonic scalpel and the decision to leave the PD open proved to be safe, effective and minimally invasive.

## Consent

Written informed consent was obtained from the patient for publication of this case report and any accompanying images. A copy of the written consent is available for review by the Editor in Chief of this journal.

## Abbreviations

**BC: **bronchogenic cysts; **PD: **pericardial defect; **VATS: **video-assisted thoracoscopic surgery; **CT: **Computed tomography.

## Competing interests

The authors declare that they have no competing interests.

## Authors' contributions

All Authors: 1. have made substantial contributions to conception and design, or acquisition of data, or analysis and interpretation of data; 2. have been involved in drafting the manuscript or revising it critically for important intellectual content; 3. have given final approval of the version to be published.
